# Pulmonary blood volume as a marker of adenosine‐induced cardiac hyperemia: A Rubidium‐82 study

**DOI:** 10.1111/cpf.70001

**Published:** 2025-02-26

**Authors:** Martin Lyngby Lassen, Jacob Peter Hartmann, Christina Byrne, Ronan M. G. Berg, Andreas Kjær, Philip Hasbak

**Affiliations:** ^1^ Department of Clinical Physiology and Nuclear Medicine University Hospital Copenhagen – Rigshospitalet Copenhagen Denmark; ^2^ Department of Biomedical Sciences Faculty of Health and Medical Sciences, Cluster for Molecular Imaging University of Copenhagen Copenhagen Denmark; ^3^ Department of Clinical Medicine Faculty of Health and Medical Sciences Copenhagen Denmark; ^4^ Centre for Physical Activity Research University Hospital Copenhagen – Rigshospitalet Copenhagen Denmark; ^5^ Neurovascular Research Laboratory Faculty of Life Sciences and Education University of South Wales Cardiff UK

**Keywords:** 82‐Rubidium, adenosine, cardiac PET, myocardial flow reserve, pharmacological stress, pulmonary blood volume

## Abstract

**Purpose:**

To investigate the efficacy of pulmonary blood volume (PBV) as a marker of the cardiac hyperemic response to adenosine during myocardial perfusion imaging (MPI).

**Methods:**

Forty healthy subjects underwent four consecutive Rubidium‐82 rest/adenosine‐stress MPI: two sessions were conducted without any caffeine consumption (baseline), while the remaining two sessions involved controlled caffeine consumption (arm 1: 100 and 300 mg; arm 2: 200 and 400 mg). We evaluate the ability of the stress‐to‐rest ratio of PBV (PBV ratio) to identify an adequate cardiac hyperemic response. The adequate hyperemic response was defined as a stress myocardial blood flow >2 mL/g/min and a corresponding myocardial flow reserve >68% of the maximum myocardial flow reserve obtained during the baseline scans.

**Results:**

Based on 126 MPI sessions conducted in 40 subjects, the PBV ratio demonstrated a sensitivity of 78% and a specificity of 74% in detecting adequate cardiac hyperemia. The positive predictive value was 95%, while the negative predictive value was 36%.

**Conclusion:**

The PBV ratio permits the identification of adequate hyperemic response with sensitivities and specificities comparable to existing markers.

## 1 INTRODUCTION

1.1

Rubidium‐82 (^82^Rb) myocardial perfusion imaging (MPI) plays a crucial role in the noninvasive diagnostic evaluation of patients suspected of having ischemic heart disease, often employing rest/stress imaging protocols (Bateman et al., 2016; Dilsizian et al., 2016). However, the accuracy of stress myocardial blood flow and flow reserve estimates is significantly compromised when pharmacologically induced cardiac hyperemia is inadequate (Rischpler and Totzeck, 2019). Elevated plasma caffeine concentrations, among other factors, may impede the vasodilator effect of adenosine, which is a common challenge in clinical practice due to the widespread use of caffeine in the general population (Lassen, Byrne, et al., 2022; Lassen, Wissenberg, et al., 2022). Consequently, there is a pressing need for a reliable marker of maximal cardiac hyperemic response that can be applied in the diagnostic assessment of MPI PET (Lassen, Byrne, et al., 2022). Previous studies have proposed using several physiological and image‐derived markers to identify the sufficient hyperemic response, of which none serves as an ideal marker. This study aims to test the feasibility of using cardiac ^82^Rb‐PET to estimate the pulmonary blood volume (PBV) as a marker for a sufficient hyperemic response (Lassen et al., 2023). The PBV depends on cardiac and pulmonary function, which the baroreceptor system monitors. Therefore, changes in homeostasis, such as during the sufficient hyperemic response to adenosine stressing, are expected to affect the pulmonary blood volume (Lassen et al., 2023).

## 2 MATERIALS AND METHODS

### 2.1 Study population

Forty healthy subjects (median age of 23 y [Inter quartile range: 22; 25 y]) participated in this study. They underwent repeated ^82^Rb MPI on a Siemens Biograph mCT system, with and without controlled caffeine intake, before the imaging sessions (Lassen et al., 2022a). The inclusion criteria comprised individuals aged >18 years who had not participated in studies testing drugs, had no regular medication intake, had no known medical conditions, and had abstained from tobacco and euphoric substances (excluding alcohol) for 3 months before the study. Exclusion criteria included pregnancy, allergies, intolerance to theophylline or adenosine, previous history of asthma, and an inability to adhere to the study protocol. The study was approved by the Scientific Ethics Committee of the Capital Region of Denmark [protocol number H‐15009293], and all participants provided informed oral and written consent. The Danish Data Protection Agency approved this study.

### 2.2 Imaging protocol

#### 2.2.1 Positron emission tomography acquisition

The detailed acquisition protocol was described in a previous study conducted at our center (Lassen et al., 2022a). Subjects underwent four MPI sessions, consisting of rest/adenosine stress MPI. Two sessions were performed with controlled caffeine consumption, where subjects orally ingested caffeine tablets diluted in hot water. The controlled caffeine intake was divided into two arms: arm 1 with doses of 100 and 300 mg and arm 2 with doses of 200 and 400 mg. Caffeine consumption occurred 1 h before the MPI sessions. Additionally, two baseline scans were conducted without caffeine intake (Lassen et al., 2022a). Of importance, the order of the repeated scans were randomized. The plasma caffeine concentration was evaluated for each scan during the stress MPI by averaging measurements obtained at 75 and 90 min post‐ingestion of caffeine. All subjects were instructed to abstain from caffeine for at least 24 h before each MPI session. All measurements were obtained using high‐performance liquid chromatography–mass spectrometry (LC‐MS/MS).

**Figure 1:**
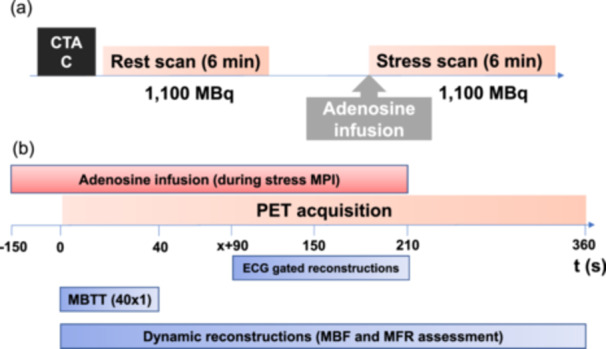
MPI protocol and image reconstructions. (a) MPI protocol for each of the four ^82^Rb‐PET/CT MPI sessions. (b) Pictogram illustrating the image reconstructions utilized. In b), the three different reconstructions employed for evaluating the ability of the PBV ratio to serve as a marker of adequate hyperemia are depicted. CTAC, CT attenuation correction; MBF, myocardial blood flow; MBTT, Mean bolus transit time; MFR, Myocardial flow reserve; PET, Positron Emission Tomography.

Each acquisition had a targeted injection dose of 1100 MBq (30 mCi) ^82^Rb (Figure 1a). Pharmacological stress was induced using adenosine infused at a rate of 140 µg/kg/min for 6 min, with the emission acquisition starting 2.5 min into the infusion (Figure 1b).

#### 2.2.2 Positron emission tomography reconstruction protocol and data processing

For each MPI acquisition, a total of three imaging series were analyzed (Figure 1b); two dynamic imaging series and an 8‐frame‐ECG gated image reconstruction. The first dynamic imaging series consisted of 18 frames (1 × 10 s, 8 × 5 s, 3 × 10 s, 2 × 20 s, 4 × 60 s) used for assessing myocardial blood flow (MBF) and flow reserve (MFR) (Byrne et al., 2021). The second dynamic imaging series included 40 frames (40 × 1 s) and was used to evaluate the mean bolus transit time (MBTT) (Lassen et al., 2023). The 8‐frame‐ECG gated reconstruction followed an optimized reconstruction protocol employing data obtained from the ^82^Rb bolus arrival in the heart +90 s until 210 s into the scan to ensure accurate LVEF assessment during adenosine‐induced hyperemia (Lassen, Wissenberg, et al., 2022). All reconstructions employed an iterative 3D ordered subset expectation maximization model with corrections for time‐of‐flight and point‐spread utilizing 2 iterations and 21 subsets. Myocardial blood flow was calculated using the Lortie model (Lortie et al., 2007). All analyses, including myocardial blood flows, flow reserves, and volumetric assessments, were conducted using a dedicated cardiac software toolbox (QPET, Cedars‐Sinai Medical Center).

#### 2.2.3 MFR: Normal range and reproducibility

As described in our previous study, the healthy subjects were expected to have normal MFR ( ≥ 2.5); thus, the usual thresholds for normal flow reserve could not be used (Lassen et al., 2022a; Murthy et al., 2018). To ameliorate the anticipated increased MFR values, we introduced a population‐based cut‐off level based on the hyperemic responses obtained in patients with both repeat baseline scans (0 mg caffeine ingested before the scan) having plasma caffeine concentration (PCC) < 1.0 mg. For these subjects, we normalized the repeated MFR values to the intra‐subject highest MFR obtained for the baseline scans. Using only interscan variation values, we obtained the normal interscan variation in MFR defined as the average interscan difference for the baseline MPIs – 1 SD (average offset = 83.0%, SD = 15.0%; cut‐off = 68%), similar to a method proposed by Bami et al. (Bami et al., 2019). In this context, the sufficient hyperemic response was only determined with stress myocardial blood flow >2 mL/g/min and MFR ≥ 68% of the individual maximum obtained MFR during the baseline scans. Of note is that the PCC was obtained by averaging two measurements obtained 75‐ and 90‐min post‐ingestion. Both blood samples were analyzed in high‐performance liquid chromatography‐mass spectrometry (Lassen et al., 2022a).

#### 2.2.4 PBV

PBV was assessed by measuring the mean transit time of the ^82^Rb‐bolus from the pulmonary trunk [VOI = 10 × 10 × 10 mm^3^] to the left atrium [VOI = 15 × 15 × 15 mm^3^]. The bolus arrival was determined based on a median blood pool activity exceeding 100 kBq (Lassen et al., 2023). PBV was calculated using the following formula:

(1) PBV=SV×HR×MBTT

where SV is the stroke volume, HR is the heart rate, and MBTT is the mean bolus transit time. The empirical PBV measures were normalized for the body‐surface area (BSA) using the DuBois equation and normalized to 1.73 m^3^ (Lassen et al., 2023).

### 2.3 Statistical analysis

The data were analyzed using R (The GNU project). Descriptive statistics were employed as appropriate, including mean ± standard deviation or median and interquartile range. Statistical comparisons between measures obtained with plasma caffeine concentration (PCC) < 1 mg/L and PCC ≥ 1 mg/L were conducted using two‐way *t*‐tests, while differences in PBV measures and MFR were obtained using Kruskal‐Walis tests for median values. Diagnostic rates, including sensitivity, specificity, and the positive and negative predictive values of the PBV ratio in detecting maximal hyperemic response, were calculated using the conventional 2 × 2 confusion matrix notation (Trevethan, 2017).

## 3 RESULTS

Out of the 160 MPI sessions initially acquired, 31 sessions were excluded from the study due to technical issues or noncompliance with the imaging protocol, as previously described (Lassen et al., 2022a). Additionally, three MPI sessions were excluded as the bolus arrival in the right ventricle occurred too early due to a delayed start of the PET acquisition. Therefore, a total of 126 MPI sessions were included and analyzed in this study.

**Figure 2:**
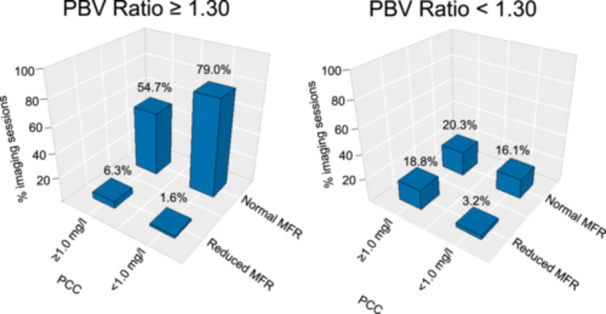
Bar plots of studies identified with and without adequate hyperemic response identified by PBV ratio stratified by PCC. An adequate hyperemic response was defined as a PBV ratio ≥ 1.30. The numbers displayed above the bars represent the percentage of occurrences for each respective category. MFR, myocardial flow reserve; PBV ratio: ratio from stress to rest pulmonary blood volume; PCC, plasma caffeine concentration.

**Figure 3:**
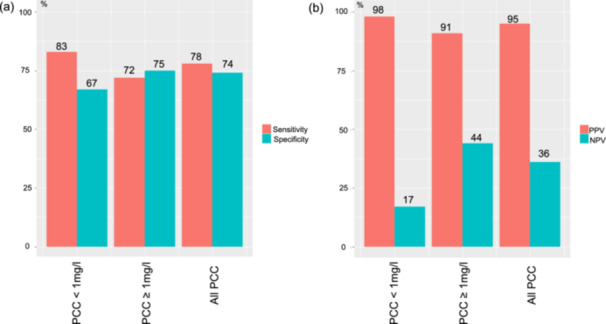
Diagnostic rates of PBV ratio for detecting adequate cardiac hyperemia. (a) displays the sensitivity and specificity of the PBV ratio for MPI scans with and without elevated PCC, as well as combined measures. (b) illustrates the PPV and NPV using the same PCC stratification. PBV ratio, ratio from stress to rest pulmonary blood volume, PCC, Plasma caffeine concentration.

A discrepancy was noted between the number of MPI sessions exhibiting adequate cardiac hyperemic response and the overall number of MPI sessions conducted (Table 1). Caffeine consumption influenced the measures of PBV; an inverse correlation is observed between the ingested dose of caffeine and both stress PBV and PBV ratios. However, the observed correlation did not reach statistical significance when comparing scans associated with varying levels of caffeine intake (Table 2). Assessments of the PBV ratio, when grouped into PCC < 1 mg/L and PCC ≥ 1 mg/L, however, revealed that caffeine consumption before the scans significantly reduces the PBV ratio (Table 1). An optimal threshold for the identification of the sufficient hyperemic response was found to be 1.30, using receiver operating curves. Utilizing this threshold, the most favorable performance was observed when PCC < 1.0 mg/L with correct identification in more than 75% of the MPI sessions. Figure 2 presents a bar plot illustrating the distribution of sessions with and without adequate hyperemic response, as determined by the PBV ratio. Diagnostic rates can be found in Figure 3. Scans with PCC < 1 mg/L demonstrated the highest sensitivity (83%), whereas scans with PCC ≥ 1.0 mg/L exhibited the best specificity (75%). A similar pattern was observed for PPV and NPV, respectively.

**Table 1:** MPI measurements and performance of PBV ratio in assessing adequate cardiac hyperemia.

	<1.0 mg/L	≥1.0 mg/L	*p*
Number of subjects (MPI sessions)	38 (62)	38 (64)	N/A
MFR	3.76 [3.39; 4.34]	3.65 [2.73;4.29]	*p* = 0.068
PCC (mg/l)	0.10 [0.05; 0.33]	**4.94 [2.86; 8.19]**	***p*** < **0.001**
PBV Ratio	1.61 [1.35; 1.88]	**1.53 [1.21; 1.69]**	***p*** = **0.042**

MPI with adequate cardiac hyperemia			
	<1.0 mg/L	≥1.0 mg/L	N/A
Correctly identified with PBV Ratio	48 (77.4%)	35 (54.7%)	N/A
Actual number	59 (95.2%)	48 (75.0%)	N/A

*Note*: The table presents Rb‐PET MPI measurements and the performance of the PBV ratio in detecting adequate cardiac hyperemia. Significant changes were observed in the PBV ratio for MPI sessions obtained with PCC ≥ 1.0 mg/L. The PBV ratio accurately identified 77.4% of all MPI sessions with an adequate hyperemic response when the PCC < 1.0 mg/L. Notably, the metrics displayed in bold for PCC ≥ 1 mg/L indicate a significant difference compared to the measures obtained for PCC < 1 mg/L.

Abbreviations: MFR, myocardial flow reserve; PBV ratio, ratio from stress to rest pulmonary blood volume; PCC, Plasma caffeine concentration.

**Table 2:** Empirical measures of rest, stress and PBV ratios in addition to the MFR measures.

	Rest PBV	Stress PBV	PBV Ratio
0 mg	693 [545; 804]	1108 [818; 1266]	1.61 [1.35; 1.92]
100 mg	603 [529; 753]	1112 [819; 1363]	1.72 [1.42; 1.95]
200 mg	600 [501; 689]	884 [733; 1080]	1.59 [1.37; 1.64]
300 mg	657 [524; 839]	865 [701; 1070]	1.32 [1.08; 1.54]
400 mg	718 [596; 786]	816 [757; 997]	1.25 [1.12; 1.40]

*Note*: All metrics are given as median [IQR]. All metrics obtained from scans following caffeine intake were significantly different from the baseline scans, while no differences were observed across the scans following caffeine intake.

## 4 DISCUSSION

The main finding of this study is that the PBV ratio (stress/rest PBV) during MPI can serve as a readily accessible index of the maximal cardiac hyperemic response to adenosine under controlled PCC conditions. The sensitivities and specificities of PBV were comparable to the most effective among the seven previously tested markers for adequate hyperemic response (splenic intensity ratio, the splenic response ratio, changes (empirical and percentwise) in the heart rate, changes in the rate‐pressure product, the systolic blood pressure, and cardiovascular resistance) (Lassen et al., 2022a). Although the PBV ratio did not outperform the existing markers, we suggest that PBV could be incorporated into the clinical routine as an additional measure or combined with current metrics to assess adequate hyperemic response.

In this study, and in a previous study from our group (Lassen et al., 2022a), we demonstrated that adenosine stress increases the PBV in healthy young volunteers who exhibit a sufficient hyperemic response. In this context, this study is a post hoc study evaluating PBV as a potential hyperemic marker. This analysis was not included in the previous study, given that the methodology to extract PBV from routine ^82^Rb PET imaging protocols was developed after the first publication.

An elevated PBV may be associated with certain side effects, most notably dyspnoea. The vasodilatory effects of adenosine may cause a transient imbalance in blood flow distribution, resulting in increased pulmonary congestion and a sensation of breathlessness. While these side effects are reversible upon discontinuation of the adenosine infusion, they should be closely monitored by healthcare professionals conducting the stress test.

The accuracy of stress MBF and MFR assessments is compromised without adequate pharmacological stressing. Studies show that 3%–20% of stress MPI may yield false‐negative results due to inadequate stressing (Kidambi et al., 2016; Rischpler and Totzeck, 2019; Sampson et al., 2007). Caffeine, and other methylxanthines may reduce the sensitivity of noninvasive myocardial imaging. Identifying an adequate hemodynamic response is challenging, but PBV may assist.

In this study, the adequate hyperemic cardiac response was deliberately hindered by administering escalating doses of caffeine (100–400 mg) before the MPI sessions (Lassen et al., 2022a). The ingestion of caffeine 1 h before the MPI session resulted in decreased MFR and PBV ratio values (Table 1). Notably, the alteration in PBV ratio exhibited favorable sensitivities and specificities in determining the attainment of an adequate maximal cardiac hyperemic response, as evidenced by excellent PPV values (Figure 3). These findings strongly support the use of the PBV ratio as a reliable indicator of maximal cardiac hyperemia during adenosine‐induced stress.

### 4.1 Limitations

This study employed PBV measures that have not yet been incorporated into routine protocols, which is a limitation. Current reconstruction toolboxes, protocols, and analytical toolboxes do not facilitate these reconstructions as standard procedures, restricting their use to centers specifically interested in evaluating the PBV and its potential applications. This study included a relatively small cohort size due to the necessity of many repeat scans, which is another limitation.

Despite the limited number of participants, the results are consistent with a previous report (Lassen et al., 2022a). However, as the study primarily included healthy volunteers, it was not possible to assess whether the PBV ratio may have any diagnostic or clinical impact on the elderly patient groups typically tested in the clinical setting, such as those with coronary artery disease. Therefore, it is not possible to draw firm conclusions on the effect of caffeine on these patient groups. This variation between younger, healthy cohorts and elderly patients who undergo ^82^Rb‐MPI for diagnostic purposes further underscores the need for caution when extrapolating these results to different patient populations.

## 5 CONCLUSION

In young, healthy volunteers, the PBV ratio can effectively indicate adequate cardiac hyperemic response when PCC is low. However, it is important to exercise caution when interpreting the PBV ratio in cases where PCC is high, as its diagnostic performance aligns closely with other indices of maximal cardiac hyperemia.

## AUTHOR CONTRIBUTIONS

**Martin Lyngby Lassen**: Conceptualization; methodology; formal analysis; investigation; data curation; writing draft; writing—review and editing; visualization. **Jacob Peter Hartmann**: Conceptualization; validation; investigation; writing draft; writing—review and editing. **Christina Byrne**: Validation; formal analysis; investigation; writing—review and editing. **Ronan M. G. Berg**: Conceptualization; methodology; investigation; writing draft; writing—review and editing. **Andreas Kjær**: Conceptualization; investigation; writing—review and editing; funding acquisition. **Philip Hasbak**: Methodology; formal analysis; investigation; writing draft; writing—review and editing; visualization.

## CONFLICT OF INTEREST STATEMENT

The authors declare no conflicts of interest.

## Data Availability

The authors have nothing to report.
